# The p21-Activated Kinase (PAK) Family Member PakD Is Required for Chemorepulsion and Proliferation Inhibition by Autocrine Signals in *Dictyostelium discoideum*

**DOI:** 10.1371/journal.pone.0096633

**Published:** 2014-05-05

**Authors:** Jonathan E. Phillips, Richard H. Gomer

**Affiliations:** Department of Biology, Texas A&M University, College Station, Texas, United States of America; University of Birmingham, United Kingdom

## Abstract

In *Dictyostelium discoideum*, the secreted proteins AprA and CfaD function as reporters of cell density and regulate cell number by inhibiting proliferation at high cell densities. AprA also functions to disperse groups of cells at high density by acting as a chemorepellent. However, the signal transduction pathways associated with AprA and CfaD are not clear, and little is known about how AprA affects the cytoskeleton to regulate cell movement. We found that the p21-activated kinase (PAK) family member PakD is required for both the proliferation-inhibiting activity of AprA and CfaD and the chemorepellent activity of AprA. Similar to cells lacking AprA or CfaD, cells lacking PakD proliferate to a higher cell density than wild-type cells. Recombinant AprA and CfaD inhibit the proliferation of wild-type cells but not cells lacking PakD. Like AprA and CfaD, PakD affects proliferation but does not significantly affect growth (the accumulation of mass) on a per-nucleus basis. In contrast to wild-type cells, cells lacking PakD are not repelled from a source of AprA, and colonies of cells lacking PakD expand at a slower rate than wild-type cells, indicating that PakD is required for AprA-mediated chemorepulsion. A PakD-GFP fusion protein localizes to an intracellular punctum that is not the nucleus or centrosome, and PakD-GFP is also occasionally observed at the rear cortex of moving cells. Vegetative cells lacking PakD show excessive actin-based filopodia-like structures, suggesting that PakD affects actin dynamics, consistent with previously characterized roles of PAK proteins in actin regulation. Together, our results implicate PakD in AprA/CfaD signaling and show that a PAK protein is required for proper chemorepulsive cell movement in *Dictyostelium*.

## Introduction

Despite substantial progress, much remains to be understood about how the size of a tissue is established during development and then subsequently maintained [1]. One mechanism of tissue size regulation involves proliferation-inhibiting factors that are secreted by, and act on, cells within a specific tissue. As the tissue size increases, levels of the factors concomitantly increase, providing negative feedback on tissue size by inhibiting proliferation in a concentration-dependent manner [2]. Such tissue specific, proliferation-inhibiting signals have been termed “chalones” [3]. Bona fide examples of chalones include myostatin, which regulates skeletal muscle size [4], [5], and GDF11, which regulates neuron number in the olfactory epithelium [6], [7]. In addition, some tumors and their metastases seem to secrete and respond to such factors, establishing a dormant state when a large tumor is established [8]. Understanding these factors and how they function might provide insight on ways to induce a dormant state in tumors and how to repress the proliferation of dormant metastases following surgical removal of a primary tumor.

We found that the secreted proteins AprA and CfaD function as chalones in the social amoeba *Dictyostelium discoideum* [9], [10]. AprA, a protein with little similarity to known human proteins, and CfaD, a member of the conserved family of cathepsin L proteases [10] are secreted by proliferating *Dictyostelium* cells and inhibit their proliferation [9], [10]. Cells lacking either AprA or CfaD proliferate more rapidly than wild-type cells and reach a higher stationary density. The addition of recombinant AprA (rAprA) or rCfaD slows the proliferation of cells. AprA shows saturable binding to cells [11], causes GTP uptake at the cell membrane [12], and requires the G proteins Gα8 and Gβ for activity [12], suggesting that AprA signals through a G protein-coupled receptor. AprA and CfaD require each other for activity [10], and also require the kinase QkgA [13], the phosphatase CnrN [14] and the putative transcription factor BzpN for activity [15]. In addition to its proliferation-inhibiting activity, AprA, but not CfaD, acts as an autocrine chemorepellent that functions to disperse groups of cells that are at high density [16]. This chemorepellent activity requires Gα8, QkgA, and CnrN but not BzpN, suggesting that AprA affects proliferation and cell movement through partially overlapping pathways.

p21-activated kinases (PAKs) are a conserved family of kinases that bind to and are activated by small GTPases such as Rac and cdc42 [17]. PAKs function to regulate actin dynamics in processes such as bud growth in *Saccharomyces cerevisiae* [18], growth cone guidance in developing *Drosophila* neurons [19] and chemotaxis towards cAMP in *Dictyostelium* [20], [21], [22]. PAK1 induces the formation of filopodia and membrane ruffles in human fibroblasts [23], whereas *Drosophila* Pak3 inhibits lammelipodia formation in cell culture [24], indicating that PAKs can positively or negatively regulate actin-based structures. PAKs also regulate proliferation [17]. In COS-1 fibroblasts, PAK1 stimulates mitogenic MAP kinase signaling [25] and in human fibroblasts, PAK2 inhibits the tumor suppressor NF2 by phosphorylation, resulting in an increase in proliferation [26]. In contrast, *Xenopus* Pak1 acts to arrest cells at mitotic metaphase during embryogenesis [27], and *Xenopus* Pak3 arrests the cell cycle and promotes neuron differentiation during neurogenesis [28]. These results indicate that depending on the context, PAKs can promote or inhibit proliferation.

PakD is a putative *Dictyostelium* PAK kinase that is involved in the regulation of F-actin during development [22]. PakD is required for aggregation during development and is required for a normal actin polymerization response to the chemoattractant cAMP. In starved cells, PakD localizes to cell extensions and to subcellular punctum structures [22]. In this report, we show that PakD negatively regulates proliferation during vegetative growth. At low cell densities, *pakD^–^* cells proliferate at the same rate as wild-type cells, but *pakD^–^* cells reach a higher maximum cell density than wild-type cells. PakD is required for the proliferation-inhibiting activity of both AprA and CfaD. Further, PakD is required for the chemorepellent effect of AprA, and *pakD^–^* cells show an increase in the size of filopodia, suggesting a role for PakD in the regulation of actin dynamics. Our data suggest that PakD is a regulator of proliferation and cell movement that functions downstream of AprA and CfaD.

## Materials and Methods

The strains Ax2 (wild-type), *pakD^–^* [22], *pakD^–^/act15::PakD-GFP, act15::PakD-GFP*, and *plc^–^* (DBS0236793, [29]) were grown in axenic shaking culture as described previously [16]. Proliferation curves, rAprA and rCfaD inhibition assays, measurement of mass, protein, and nuclei per cell, measurement of colony diameter on bacterial lawns, and measurement of proliferation on bacterial lawns were done as described previously [13]. Measurement of AprA and CfaD in conditioned media was done as described previously [13], except that conditioned media was collected from cells at a density of 1×10^7^ cells/ml. Chemorepellent assays were done as previously described [16]. The data for wild-type response to the chemorepellent activity of rAprA is identical to that published previously [16], as the previously reported data and the data presented in this paper were generated concurrently.

To construct a PakD-GFP transgene, two partially overlapping fragments of the PakD open reading frame were amplified by PCR from vegetative stage *Dictyostelium* cDNA using the primer pairs GGAGATCTATGAGTAGATTACAACCTCAACAACAACAAAGAG, CACTCTTTGATAATCCCCAACTTGC and GGAGATCTAAAATTAAAATTAATATCAGAGAATTGATTTTTACG, TCAGATTATGATAAAGATATGGTAGATTTTGG. Respectively, these primer pairs generate a 5′ PakD gene fragment with a BglII site preceding the start codon, and a 3′ PakD gene fragment with a BglII site immediately following the codon encoding the terminal amino acid and thus replacing the stop codon. Both gene fragments contain an overlapping region of the PakD gene that contains a SpeI site. These two fragments were independently cloned into pGEM-T Easy vectors (Promega, Madison, Wisconsin). The cloned gene fragments were then digested with BglII and SpeI, gel purified, and used in a three-part ligation with the extrachromosomal vector pDM323 [30] digested with BglII, resulting in a vector encoding a complete PakD gene with GFP fused to the C-terminus. Correct sequence and proper gene orientation in the vector were confirmed with DNA sequencing of the entire PakD gene and the restriction enzyme site junctions. This extrachromosomal vector was then transformed into *Dictyostelium* cells using standard electroporation protocols [31].

To image PakD-GFP localization by deconvolution microscopy, spots of *actin15::PakD-GFP* cells were grown in a 1.5 ml volume of HL5 in 2-well glass chamber slides (Nunc) overnight, and cells were subsequently fixed and stained with DAPI as described previously [15]. Cells were then imaged using an Olympus FV1000 microscope with a 100×1.2 NA objective, and image z-stacks were generated with a slice separation of 0.2 microns. Z-stacks were then processed using Autodeblur deconvolution software (Bitplane software, Zurich, Switzerland). To stain cells with Alexa Fluor 594 Phalloidin (Invitrogen, Carlsbad, CA), cells were fixed as described above and then stained with phalloidin as previously described [32]. To label the centrosome in cells expressing PakD-GFP, spots of *actin15::PakD-GFP* cells were grown in glass chamber slides overnight, and cells were then fixed for 30 minutes with 4% paraformaldehyde in PHEM buffer (30 mM Na-PIPES, 12.5 mM HEPES, 5 mM EGTA, 1 mM MgCl_2_, pH 6.9 [33]). Cells were washed three times in PBS and permeablized in PBS with 0.1% NP-40 for 10 minutes. Cells were then stained with anti-DdCP224 antibodies as previously described [34]. Cells were then mounted in Vectashield mounting media with DAPI (Vector, Burlingame, CA) and imaged as described above. To image PakD-GFP in live cells, spots of *pakD^–^/act15::PakD-GFP* cells were grown in 2-well glass chamber slides (Nunc) overnight in FM media (Formedium, Norwich, UK). Cells were then imaged using an Olympus FV1000 confocal microscope with a 100× objective by time-lapse microscopy. All statistical analyses were done with Prism (GraphPad Software, San Diego, CA). Significance was defined as a p value of <0.05.

## Results

### PakD negatively regulates cell proliferation

**Figure 1:**
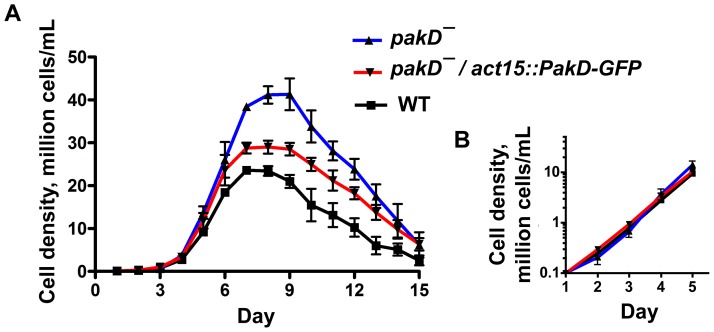
Cells lacking PakD proliferate to a higher density than wild-type cells. (**A**) Cells were inoculated into axenic shaking culture at 1×10^5^ cells/ml and counted daily. (**B**) The data from days 1–5 were plotted using a log scale. Values are mean ± SEM, n≥3. The absence of error bars indicates that the SEM is smaller than the plot symbol.

Kinases of the p21-activated kinase (Pak) family are involved in signal transduction pathways regulating processes such as cell motility [24] and proliferation [27]. PakD is a 190 kD PAK protein in *Dictyostelium* with putative kinase, diacylglycerol-binding, Cdc42/Rac interactive binding (CRIB), and calponin-homology (which functions in actin binding [35]) domains. Because AprA and PAKs affect both proliferation and motility, we examined whether PakD plays a role in AprA/CfaD signaling. Cells lacking AprA or CfaD show a faster doubling time than wild-type cells during logarithmic growth and reach a higher stationary density than wild-type cells [9], [10]. We found that *pakD^–^* cells showed a doubling time like wild-type cells during the logarithmic growth phase, but that *pakD^–^* cells proliferated to a significantly higher cell density than wild-type cells, and that this phenotype could be rescued by expression of a PakD-GFP transgene (Figure 1 and Table 1). Cells lacking AprA and CfaD show a rapid decrease in cell density after reaching stationary density in shaking culture [9], [10], suggesting that these proteins increase cell viability after stationary density has been reached. However, *pakD^–^* cells did not show this rapid decrease in cell density after stationary density had been reached (Figure 1A), suggesting that PakD does not significantly affect the viability of cells at high density. These results indicate that PakD inhibits proliferation at high cell density and that *pakD^–^* cells exhibit some but not all of the phenotypes of *aprA^–^* and *cfaD–* cells.

**Table 1:** The effect of PakD on the doubling time and stationary density of cells.

Genotype	Doubling time, hours	Maximum observed cell density, 10^6^ cells/mL
Wild type	13.6±0.3	24.3±0.7
*pakD^–^*	12.0±0.6	43.6±2.7***
*pakD^–^/actin15::pakD-GFP*	13.7±0.6	29.3±1.3

Doubling times and stationary densities were calculated for the proliferation curves in Figure 1. Values are mean ± SEM from three independent experiments. “***” indicates that the difference between the value and the wild-type value is significant with p<0.001 (one-way ANOVA, Tukey's test). The difference between the maximum observed density between *pakD^–^* and *pakD/pakD-GFP* is significant (p<0.01, Tukey's test).

**Figure 2:**
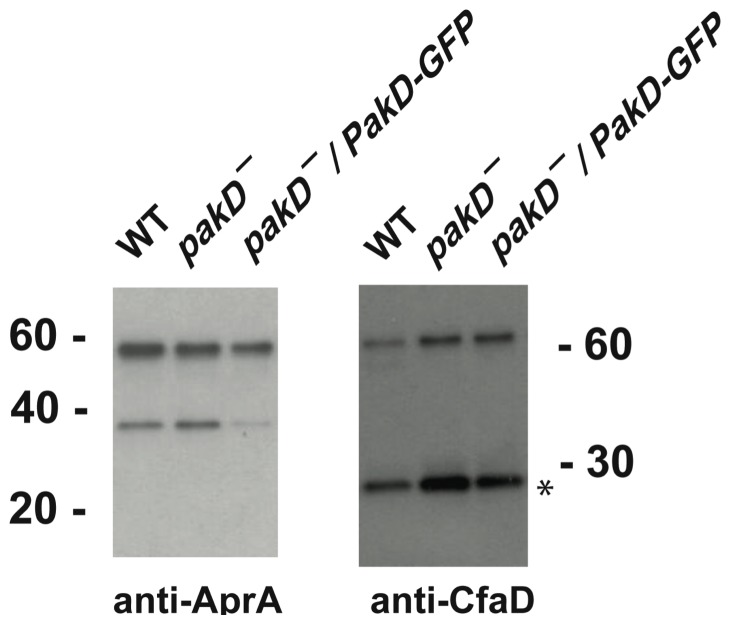
*pakD^–^* cells accumulate extracellular AprA and CfaD. Conditioned media from cells at 1×10^7^ cells/ml was collected and stained for AprA and CfaD by Western blots. The asterisk indicates a 27-kDa breakdown product of CfaD. The non-specific band at 35 kDa in the AprA blot varied in intensity between experiments and between genotypes.

As AprA and CfaD are extracellular signals that slow proliferation and increase in concentration at high cell density, one explanation for an increased proliferation phenotype is a lack of extracellular AprA or CfaD. To test this possibility, we examined the levels of AprA and CfaD in high-density conditioned media from *pakD^–^* cells by Western blots. *pakD^–^* and *pakD^–^/act15::PakD-GFP* cells showed extracellular accumulation of AprA and CfaD similar to or higher than that of wild type (Figure 2). These results strongly suggest that the high cell density of *pakD^–^* cells is not due to a lack of AprA or CfaD.

**Figure 3:**
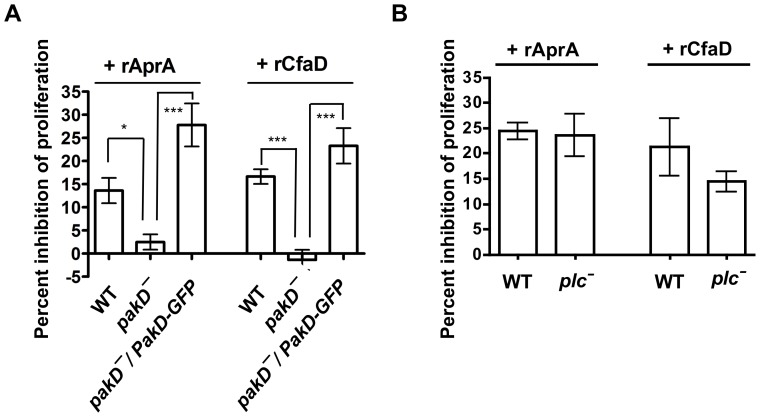
*pakD^–^* cells are insensitive to proliferation inhibition by AprA and CfaD. (**A**) Log-phase cells were collected, resuspended to 0.5×10^6^ cells/ml, and either 300 ng/ml rAprA, 150 ng/ml rCfaD, or an equivalent volume of buffer was added to the cell culture. The inhibition of proliferation after 16 hours as a percent of the buffer control is plotted (100– ((experimental cell density/control cell density) * 100%)). “*” indicates that the difference in values between the conditions are significant with p<0.05, and “***” indicates p<0.001 (One-way ANOVA, Tukey's test). Values are mean ± SEM, n≥3. For both rAprA and rCfaD, the values for *pakD^–^* cells are not significantly different from a value of zero (p>0.05, paired t-test). For rAprA, but not for rCfaD, the differences between WT and *pakD^–^/PakD-GFP* are significant (p<0.05, One-way ANOVA, Tukey's test). (**B**) Wild-type and *plc^–^* cells were assayed for sensitivity to the proliferation-inhibiting activity of AprA and CfaD as described above. Differences between wild-type and *plc^–^* values are not significant (p>0.05, t-test).

Recombinant AprA (rAprA) and CfaD (rCfaD) slow the proliferation of wild-type cells [9], [10]. To determine whether PakD plays a role in AprA- and/or CfaD-mediated inhibition of proliferation, we examined the effects of rAprA and rCfaD on *pakD^–^* cells. As observed previously, wild-type cells showed reduced proliferation in response to rAprA and rCfaD (Figure 3A). In contrast, *pakD^–^* cells showed no significant reduction in proliferation in the presence of either rAprA or rCfaD. rAprA and rCfaD slowed the proliferation of *pakD^–^/act15::PakD-GFP* cells, showing that the insensitivity of the *pakD^–^* cells is due specifically to the absence of the *pakD* gene. As PakD has a predicted diacylglycerol (DAG)-binding domain [36], and phospholipase C (PLC) enzymatically generates DAG, we tested whether PLC might signal through PakD to inhibit proliferation. The proliferation of *plc^–^* cells was inhibited by rAprA and rCfaD to the same degree as wild-type cells (Figure 3B), indicating that AprA and CfaD do not signal through PLC to affect PakD. Together, these results indicate that PakD is a negative regulator of proliferation and that PakD is necessary for proliferation inhibition by AprA and CfaD.

### *pakD^–^* cells show normal nuclei, mass and protein content per cell during logarithmic growth

*aprA^–^* and *cfaD^–^* cells have more nuclei per cell than wild-type cells during vegetative growth, which may be due to an increased mitotic rate [9], [10]. We examined the nuclei content of log-phase *pakD^–^* cells by DAPI staining, and found that there was no significant difference as compared to wild type (Table 2). These results indicate that PakD does not affect the multinuclearity of cells, and suggest that AprA and CfaD affect cellular nuclei content in a manner independent of PakD.

**Table 2:** The effect of PakD on the mass, protein, and nuclei content of cells.

	Per 10^7^ cells		% cells with *n* nuclei			Nuclei/100 cells	Per 10^7^ nuclei	
Genotype	Mass (mg)	Protein (mg)	1	2	3+		Mass (mg)	Protein (mg)
Wild type	6.1±0.3	0.41±0.03	71±2	23±2	6±1	134±2	4.5±0.3	0.30±0.02
*pakD^–^*	5.3±0.3	0.36±0.02	73±3	24±3	3±1	131±4	4.0±0.3	0.28±0.01
*pakD^–^/actin15::pakD-GFP*	5.6±0.6	0.43±0.03	80±5	18±5	2±1	123±5	4.6±0.6	0.35±0.03

Mass and protein content were determined as described in the Materials and Methods. Values are the mean ± SEM from three or more independent experiments. Values for *pakD^–^* or *pakD^–^/actin15::pakD-GFP* were not significantly different from wild-type values or each other for any parameter shown (p>0.05, one-way ANOVA, Tukey's test).

Cell proliferation (the increase in cell number) and cell growth (the accumulation of mass) can be regulated independently [37]. AprA and CfaD regulate cell proliferation, but not growth on a per nucleus basis [9], [10]. To determine whether PakD affects cell mass or cell growth, we first measured the mass and protein content of cells. During exponential growth, wild-type cells showed mass and protein values like those seen previously (Table 2; [13]). *pakD^–^* cells and *pakD^–^/act15::PakD-GFP* showed mass and protein content per cell and per nucleus that were not significantly different than wild-type values, indicating that PakD does not affect mass or protein content. We then estimated growth by dividing the mass and protein values by the measured doubling times during exponential growth to calculate the mass and protein accumulation per hour. Wild-type values for mass and protein accumulation were similar to those seen previously [13], and the values for mass and protein accumulation were not significantly different between wild-type, *pakD^–^*, and *pakD^–^/act15::PakD-GFP* strains on either a per cell or per nucleus basis (Table 3). These results indicate that PakD does not affect the mass or protein accumulation of cells undergoing exponential growth.

**Table 3:** The effect of PakD on the mass and protein accumulation of cells.

	Per 10^7^ cells per hour			Per 10^7^ nuclei per hour	
Genotype	Mass (mg)	Protein (µg)	Nuclei, ×10^−5^	Mass (mg)	Protein (µg)
Wild type	0.45±0.03	30±2	9.9±0.3	0.33±0.02	22±2
*pakD^–^*	0.44±0.04	30±2	10.9±0.6	0.34±0.03	23±2
*pakD^–^/actin15::pakD-GFP*	0.41±0.06	31±3	8.9±0.6	0.33±0.05	25±3

Mass and protein values from Table 2 were divided by the observed doubling time of the respective genotype. Doubling times were calculated as described in Materials and Methods. Values are the mean ± SEM from three or more independent experiments. Values for *pakD^–^* or *pakD^–^/actin15::pakD-GFP* were not significantly different from wild-type values or each other for any parameter shown (p>0.05, one-way ANOVA, Tukey's test).

### PakD localizes to a punctum within cells

**Figure 4:**
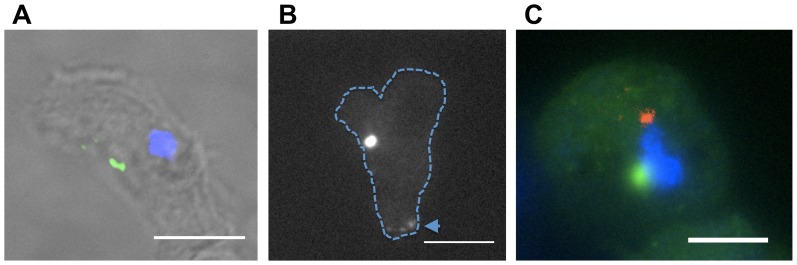
PakD tends to localize to a single punctum or at the rear of motile cells. (**A**) *act15::PakD-GFP* cells were grown in glass chamber slides overnight, then fixed and stained with DAPI (blue), imaged by fluorescence microscopy using a 100× objective, and processed using Autodeblur deconvolution software (Bitplane software, Zurich, Switzerland). PakD-GFP signal (green) is shown superimposed on a transmitted light image. Bar is 10 µm. (**B**) Live *pakD^–^/act15::PakD-GFP* cells grown as a colony in glass chamber slides were imaged by fluorescence microscopy. The imaged cell is moving upwards, as seen by time-lapse microscopy. The arrow indicates PakD-GFP at the rear of the cell. A single punctum structure is also visible. The cell border is shown with a dashed line. Bar is 10 µm. (**C**) PakD-GFP (green) does not colocalize with either the cell nucleus (blue) or with staining for an antibody against ddCP224 (Red), a marker for the centrosome. Bar is 5 µm.

We examined the subcellular localization of PakD by imaging vegetative cells expressing a PakD-GFP fusion protein. PakD-GFP localized to a punctum within the cell (Figure 4), and there tended to be one structure per cell, although occasionally more than one PakD spot per cell was observed. A similar localization of the endogenous PakD protein in vegetative cells has been seen by immunofluorescence with an antibody raised against PakD (Derrick Brazill, unpublished observations), although in starved cells PakD also localizes to cell protrusions [22]. When examining PakD-GFP in live, motile cells, a similar punctum was seen, and occasionally localization at the rear cortex of the moving cell was observed (arrow, Figure 4B and Movie S1), suggesting a role in cell movement. PakD-GFP did not co-localize with the nucleus (Figure 4A), or with anti-DdCP224 staining, a marker for the centrosome (Figure 4C, [34]). No change in PakD-GFP localization was seen in cells at high density or in cells incubated with high-density conditioned media (data not shown), suggesting that PakD localization is not altered in response to high AprA or CfaD levels. These results indicate that PakD localizes to a punctum that is not the nucleus or centrosome, and that the localization of PakD may be polarized during cell movement.

### PakD affects the ability of colonies to spread and the response of cells to the chemorepellent AprA

**Figure 5:**
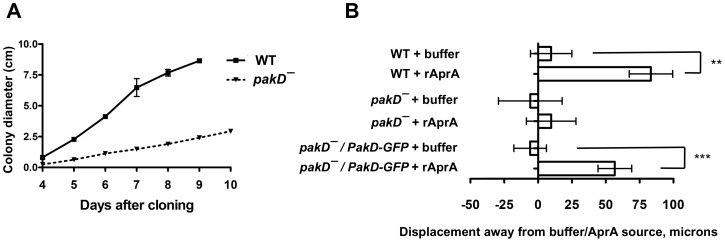
*pakD^–^* cells show reduced colony expansion and are insensitive to the chemorepellent activity of AprA. (**A**) Serial dilutions of cells were spread on SM/5 plates with bacteria, and the diameter of individual colonies was measured daily. Values are mean SEM, n = 3. The absence of error bars indicates that the SEM is smaller than the plot symbol. (**B**) Log-phase cells on glass coverslips were placed in either HL5 media alone or in HL5 media with a gradient of rAprA using an Insall chamber. Cells were filmed, and the displacement of individual cells over 1 hour in the direction of the rAprA gradient was measured. Displacement to the right of the origin indicates movement away from the rAprA source. The average displacement of cells is plotted from 3 independent experiments, with the displacements for at least 10 randomly selected cells measured for each experiment. “**” indicates p<0.01, and *** indicates p<0.001 (t-test).

AprA is an autocrine chemorepellent in vegetative *Dictyostelium* cells [16] that may facilitate the spreading out of dense groups of cells. Consistent with this model, colonies of cells lacking AprA show a reduced ability to spread on bacterial lawns [13] despite the fact that *aprA^–^* cells proliferate rapidly under these conditions [9]. To examine whether PakD may be involved in this process, we examined the size of *pakD^–^* colonies on bacterial lawns. Wild-type colonies showed a rate of expansion similar to what we observed previously [13], whereas *pakD^–^* cells showed a significantly reduced rate of expansion (Figure 5A), suggesting that PakD functions in the expansion of colonies. We then tested whether *pakD^–^* cells, like wild type cells, show directed movement away from areas of high cell density [16] by tracking cell movement at the edge of a cell colony. Under these conditions, wild-type and *pakD^–^* cells showed similar speed, whereas *pakD^–^/act15::PakD-GFP* cells were significantly slower than wild-type cells (Table 4). These results indicate that the reduced expansion of *pakD^–^* cells is not due to reduced cell speed. However, *pakD^–^* cells had a significantly reduced chemotactic index in the direction away from the cell colony as compared to wild-type cells, and this phenotype was rescued by expression of PakD-GFP in *pakD^–^* cells (Table 4). Together, these data indicate that *pakD^–^* cells show reduced ability to spread from areas of high cell density, but that this is not due to a reduced cell speed.

**Table 4:** The effect of PakD on the movement of cells at the edge of colonies.

	Cell speed (µm/minute)	Forward migration index
Wild type	6.7±0.6	0.23±0.03
*pakD^–^*	6.3±0.2	0.08±0.03**
*pakD^–^/actin15::pakD-GFP*	4.6±0.2***	0.27±0.03

Colonies of cells were established on glass chamber slides in HL5 media and, following a one-hour incubation at room temperature, cells at the edge of the colony were filmed. Videos were used to track cell movement, and the tracking data was used to calculate the given values. At least 10 randomly selected cells from each of 3 independent experiments were tracked. A positive forward migration index indicates directed movement away from the colony. “***” indicates p<0.001, and “**” p<0.01 compared to wild type (One-way ANOVA, Tukey's test).

Wild-type cells show directed movement away from a source of rAprA, indicating that AprA is a chemorepellent [16]. To test whether PakD is necessary for the chemorepellent activity of rAprA, we examined whether *pakD^–^* cells show movement away from a rAprA source. Whereas wild-type cells showed a bias in movement away from a rAprA source, *pakD^–^* cells showed no significant bias in movement, and this phenotype could be rescued by PakD-GFP expression (Figure 5B). These results indicate that PakD is necessary for rAprA chemorepellent activity.

**Figure 6:**
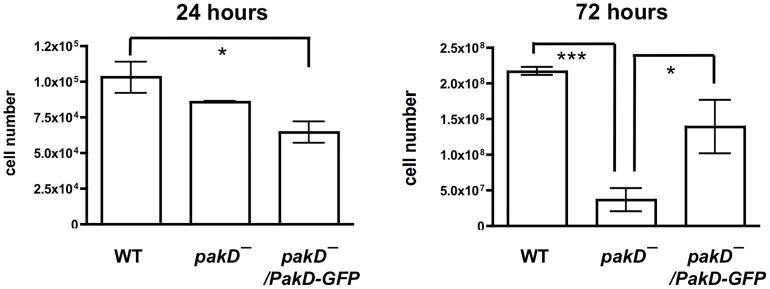
PakD affects the proliferation of cells on bacterial lawns. 1000 cells were spread with bacteria on SM/5 plates, and at the indicated times *Dictyostelium* cells were washed off the plate and counted. Values are mean ± SEM, with n = 3. “*” indicates that p<0.05, and “***” indicates that p<0.01 (One-way ANOVA, Tukey's test). At 72 hours, the difference between wild-type and *pakD^–^/PakD-GFP* is not significant.

In addition to showing rapid proliferation in shaking culture, *aprA^–^* cells proliferate rapidly when grown on bacterial lawns [9], whereas *cfaD^–^* cells do not show rapid proliferation on bacterial lawns [10]. We tested whether *pakD^–^* cells showed aberrant proliferation on lawns of bacteria by spreading 1000 cells on rich media plates with bacteria and counting the number of *Dictyostelium* cells at different time points. At 24 hours, the number of wild-type cells had increased approximately 100-fold, consistent with previous findings (Figure 6, [13]). The number of *pakD^–^* cells was not significantly different from that of wild type, though there were significantly less *pakD^–^/act15::PakD-GFP* cells at this time point, suggesting that overexpression of PakD may slow proliferation on bacteria. However, at 72 hours, there were significantly less *pakD^–^* cells than wild-type cells, and this reduced cell number was partially rescued by expression of PakD-GFP (Figure 6). At this timepoint, individual *Dictyostelium* plaques were visible on the bacterial lawns, and wild-type and *pakD^–^/act15::PakD-GFP* plaques showed more spreading than *pakD^–^* plaques. Our data suggest that, initially, PakD is not required for normal proliferation, though overexpression of PakD may slow proliferation during colony growth. However, at later time points, reduced spreading of *pakD^–^* colonies may result in reduced cell proliferation.

**Figure 7:**
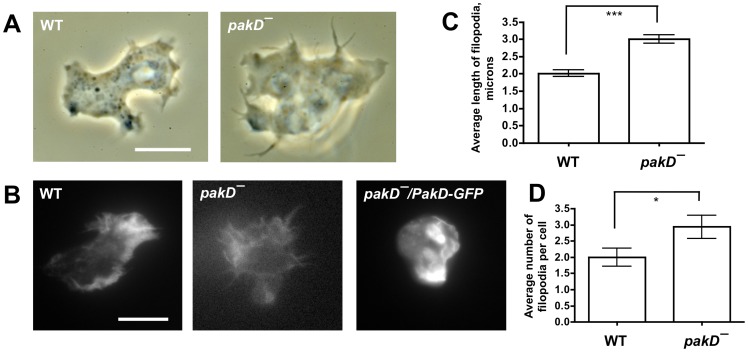
*pakD^–^* cells show enlarged filopodia. (**A**) Live vegetative cells in submerged culture were imaged using an inverted microscope with a 60× objective. Bar is 10 µm. (**B**) Cells were grown in chamber slides in HL5 media overnight and were then fixed and stained with Alexa Fluor 594 phalloidin. Expression of PakD-GFP in the shown *pakD^–^/act15::PakD-GFP* cell was confirmed by the presence of a punctum PakD-GFP signal (data not shown). Bar is 10 µm. (**C**) The filopodium length was measured for all filopodia on at least 30 cells per condition. “***” indicates p<0.001 (t-test). (**D**) The average number of filopodia per cell visible in a single focal plane was measured by counting filopodia on at least 30 cells per condition. “*” indicates p<0.05 (t-test).

PAKs regulate actin dynamics [22], [38]. To examine whether PakD is involved in the regulation of actin-based structures during vegetative growth, we examined the morphology of live cells in growth media and of fixed cells stained with phalloidin, an F-actin-binding molecule. Live, randomly motile vegetative *pakD^–^* cells showed enlarged filopodia-like extensions at the cell periphery as compared to wild-type cells (Figure 7A). Similarly, fixed *pakD^–^* cells showed enlarged spiked F-actin structures at the periphery (Figure 7B). Expression of PakD-GFP in *pakD^–^* cells resulted in a reduction of these structures (Figure 7B). Quantification of filopodia size and frequency in vegetative cells showed that *pakD^–^* cells have longer filopodia (Figure 7C) and more filopodia per cell (Figure 7D) than wild-type cells. These results suggest that PakD negatively regulates the formation of filopodia in vegetative cells.

## Discussion

The mechanisms by which chalones act to slow proliferation and thus regulate tissue size are poorly understood. Our data indicate that the kinase PakD is a negative regulator of proliferation and is necessary for the activity of the chalones AprA and CfaD, indicating that PakD is involved in chalone signaling. Like *aprA^–^* and *cfaD^–^* cells, *pakD^–^* cells accumulate mass and protein on a per nucleus basis during exponential growth at a rate like that of wild type, indicating that PakD regulates proliferation but does not significantly affect cell growth. However, *pakD^–^* cells differ in some ways from *aprA^–^* or *cfaD^–^* cells. First, whereas *aprA^–^* and *cfaD^–^* cells show rapid proliferation even at lower densities [9], [10], *pakD^–^* cells only show a difference in proliferation as compared to wild type at high densities. Second, *aprA^–^* and *cfaD^–^* cells are multinucleate, whereas *pakD^–^* cells are not. Therefore, PakD likely mediates some but not all of the affects of AprA and CfaD. As *pakD^–^* cells proliferate like wild type at lower densities, some separate branch of the signal transduction pathway must mediate the effects of AprA and CfaD for these lower density cells. This branch may involve the transcription factor BzpN, which is necessary for AprA/CfaD signaling and affects proliferation primarily at lower cell densities [15].

We found that PakD-GFP shows a punctum-like subcellular localization that tends to localize in a single spot, and that this structure is not the centrosome or part of the nucleus. This punctum structure is likely not an artifact of GFP fusion, as the native PakD protein shows a similar punctum localization in both starved and vegetative cells [22]. PakD thus may be part of the Golgi or an uncharacterized subcellular structure. Alternatively, PakD may form endogenous, functioning self-aggregates, analogous to proteins such as CPEB [39] or Orb2 [40]. In starved cells, some PakD appears to be associated with filopodia [22]. We did not observe PakD-GFP in filopodia in vegetative cells, suggesting that some of the PakD localization changes during differentiation.

PAKS are predominantly considered promoters and not inhibitors of cell growth and proliferation and are considered oncogenic in some circumstances [41]. However, PAKs function to inhibit proliferation during *Xenopus* development [27], [28]. Our data suggests that PAKs also show antiproliferative activity in *Dictyostelium*, suggesting conservation of this function. It may therefore be useful to determine whether any human PAKs show a similar function, and whether PAK activity is affected by autocrine signaling. As PAKs are regulated by Rho/Rac/cdc42-type GTPases, it will be interesting in the future to test whether such proteins negatively regulate proliferation in vertebrates or in *Dictyostelium*, which has several Rho GTPase orthologs [42].

Apart from the proliferation-inhibiting activity of PakD, we also found that PakD is necessary for the chemorepellent activity of AprA, but does not affect the average speed of cells. Further, PakD is involved in the negative regulation of actin-based structures at the cell periphery. One appealing model for chemorepulsion consists of the recruitment of active PakD to subcellular areas of high AprA signaling due to an AprA gradient. This polarized PakD activity could potentially inhibit the development of actin-based structures in directions corresponding to high AprA levels, inhibiting movement up an AprA gradient, and thus potentiating movement down an AprA gradient. This model is supported by our observation of PakD-GFP at the rear of cells, but further studies are required to test this model more rigorously.

Much remains to be understood about how endogenous chemorepellents function in eukaryotic cells. We have shown that PAKs, which have been found to play a role in Semaphorin-mediated chemorepulsion during axonal guidance in vertebrates [43], also are necessary for chemorepulsion in *Dictyostelium*. A better understanding of chemorepulsive processes could be useful for resolving inflammation [44] or for preventing the dispersion and metastasis of tumors [45]. The conservation of PAK function between *Dictyostelium* and metazoans suggests that further study of AprA-mediated chemorepulsion could reveal important, uncharacterized regulators of chemorepulsive processes.

## Supporting Information

Movie S1 **PakD-GFP localization in a motile cell.** *pakD^–^/actin15::PakD-GFP c*ells were imaged as described in Figure 4B over a period of 5 minutes using time lapse microscopy.
(MOV)

## References

[1] Halder G, Johnson RL (2011) Hippo signaling: growth control and beyond. Development 138: 9–22. 21138973 10.1242/dev.045500 PMC2998162

[2] Gomer RH (2001) Not being the wrong size. Nat Rev Mol Cell Biol 2: 48–54. 11413465 10.1038/35048058

[3] Bullough WS, Hewett CL, Laurence EB (1964) The Epidermal Chalone; a Preliminary Attempt at Isolation. Exp Cell Res 36: 192–200. 14222740 10.1016/0014-4827(64)90172-7

[4] Schiaffino S, Dyar KA, Ciciliot S, Blaauw B, Sandri M (2013) Mechanisms regulating skeletal muscle growth and atrophy. FEBS J 280: 4294–4314. 23517348 10.1111/febs.12253

[5] McPherron AC, Lawler AM, Lee SJ (1997) Regulation of skeletal muscle mass in mice by a new TGF-beta superfamily member. Nature 387: 83–90. 9139826 10.1038/387083a0

[6] Gokoffski KK, Wu HH, Beites CL, Kim J, Kim EJ, et al (2011) Activin and GDF11 collaborate in feedback control of neuroepithelial stem cell proliferation and fate. Development 138: 4131–4142. 21852401 10.1242/dev.065870 PMC3171217

[7] Wu HH, Ivkovic S, Murray RC, Jaramillo S, Lyons KM, et al (2003) Autoregulation of neurogenesis by GDF11. Neuron 37: 197–207. 12546816 10.1016/s0896-6273(02)01172-8

[8] Demicheli R (2001) Tumour dormancy: findings and hypotheses from clinical research on breast cancer. Semin Cancer Biol 11: 297–306. 11513565 10.1006/scbi.2001.0385

[9] Brock DA, Gomer RH (2005) A secreted factor represses cell proliferation in Dictyostelium. Development 132: 4553–4562. 16176950 10.1242/dev.02032 PMC4484793

[10] Bakthavatsalam D, Brock DA, Nikravan NN, Houston KD, Hatton RD, et al (2008) The secreted Dictyostelium protein CfaD is a chalone. J Cell Sci 121: 2473–2480. 18611962 10.1242/jcs.026682 PMC2716657

[11] Choe JM, Bakthavatsalam D, Phillips JE, Gomer RH (2009) Dictyostelium cells bind a secreted autocrine factor that represses cell proliferation. BMC Biochem 10: 4. 19187549 10.1186/1471-2091-10-4 PMC2644720

[12] Bakthavatsalam D, Choe JM, Hanson NE, Gomer RH (2009) A Dictyostelium chalone uses G proteins to regulate proliferation. BMC Biol 7: 44. 19635129 10.1186/1741-7007-7-44 PMC2726123

[13] Phillips JE, Gomer RH (2010) The ROCO kinase QkgA is necessary for proliferation inhibition by autocrine signals in Dictyostelium discoideum. Eukaryot Cell 9: 1557–1565. 20709790 10.1128/EC.00121-10 PMC2950431

[14] Herlihy SE, Tang Y, Gomer RH (2013) A Dictyostelium secreted factor requires a PTEN-like phosphatase to slow proliferation and induce chemorepulsion. PLoS One 8: e59365. 23555023 10.1371/journal.pone.0059365 PMC3595242

[15] Phillips JE, Huang E, Shaulsky G, Gomer RH (2011) The putative bZIP transcripton factor BzpN slows proliferation and functions in the regulation of cell density by autocrine signals in Dictyostelium. PLoS One 6: e21765. 21760904 10.1371/journal.pone.0021765 PMC3131300

[16] Phillips JE, Gomer RH (2012) A secreted protein is an endogenous chemorepellant in Dictyostelium discoideum. Proc Natl Acad Sci U S A 109: 10990–10995. 22711818 10.1073/pnas.1206350109 PMC3390837

[17] Bokoch GM (2003) Biology of the p21-activated kinases. Annu Rev Biochem 72: 743–781. 12676796 10.1146/annurev.biochem.72.121801.161742

[18] Leberer E, Dignard D, Harcus D, Thomas DY, Whiteway M (1992) The protein kinase homologue Ste20p is required to link the yeast pheromone response G-protein beta gamma subunits to downstream signalling components. EMBO J 11: 4815–4824. 1464311 10.1002/j.1460-2075.1992.tb05587.x PMC556957

[19] Hing H, Xiao J, Harden N, Lim L, Zipursky SL (1999) Pak functions downstream of Dock to regulate photoreceptor axon guidance in Drosophila. Cell 97: 853–863. 10399914 10.1016/s0092-8674(00)80798-9

[20] Chung CY, Firtel RA (1999) PAKa, a putative PAK family member, is required for cytokinesis and the regulation of the cytoskeleton in Dictyostelium discoideum cells during chemotaxis. J Cell Biol 147: 559–576. 10545500 10.1083/jcb.147.3.559 PMC2151188

[21] Lee S, Rivero F, Park KC, Huang E, Funamoto S, et al (2004) Dictyostelium PAKc is required for proper chemotaxis. Mol Biol Cell 15: 5456–5469. 15483055 10.1091/mbc.E04-04-0323 PMC532025

[22] Garcia M, Ray S, Brown I, Irom J, Brazill D (2013) PakD, a Putative p21-Activated Protein Kinase in Dictyostelium discoideum Regulates Actin. Eukaryot Cell 13: 119–126. 24243792 10.1128/EC.00216-13 PMC3910960

[23] Sells MA, Knaus UG, Bagrodia S, Ambrose DM, Bokoch GM, et al (1997) Human p21-activated kinase (Pak1) regulates actin organization in mammalian cells. Curr Biol 7: 202–210. 10.1016/s0960-9822(97)70091-5 9395435

[24] Asano Y, Jimenez-Dalmaroni A, Liverpool TB, Marchetti MC, Giomi L, et al (2009) Pak3 inhibits local actin filament formation to regulate global cell polarity. HFSP J 3: 194–203. 19639041 10.2976/1.3100548 PMC2714956

[25] Eblen ST, Slack JK, Weber MJ, Catling AD (2002) Rac-PAK signaling stimulates extracellular signal-regulated kinase (ERK) activation by regulating formation of MEK1-ERK complexes. Mol Cell Biol 22: 6023–6033. 12167697 10.1128/MCB.22.17.6023-6033.2002 PMC134005

[26] Kissil JL, Johnson KC, Eckman MS, Jacks T (2002) Merlin phosphorylation by p21-activated kinase 2 and effects of phosphorylation on merlin localization. J Biol Chem 277: 10394–10399. 11782491 10.1074/jbc.M200083200

[27] Rooney RD, Tuazon PT, Meek WE, Carroll EJ, Hagen JJ, et al (1996) Cleavage arrest of early frog embryos by the G protein-activated protein kinase PAK I. J Biol Chem 271: 21498–21504. 8702934 10.1074/jbc.271.35.21498

[28] Souopgui J, Solter M, Pieler T (2002) XPak3 promotes cell cycle withdrawal during primary neurogenesis in Xenopus laevis. EMBO J 21: 6429–6439. 12456650 10.1093/emboj/cdf644 PMC136948

[29] Keizer-Gunnink I, Kortholt A, Van Haastert PJ (2007) Chemoattractants and chemorepellents act by inducing opposite polarity in phospholipase C and PI3-kinase signaling. J Cell Biol 177: 579–585. 17517960 10.1083/jcb.200611046 PMC2064204

[30] Veltman DM, Akar G, Bosgraaf L, Van Haastert PJ (2009) A new set of small, extrachromosomal expression vectors for Dictyostelium discoideum. Plasmid 61: 110–118. 19063918 10.1016/j.plasmid.2008.11.003

[31] Gaudet P, Pilcher KE, Fey P, Chisholm RL (2007) Transformation of Dictyostelium discoideum with plasmid DNA. Nat Protoc 2: 1317–1324. 17545968 10.1038/nprot.2007.179

[32] Tang Y, Gomer RH (2008) A protein with similarity to PTEN regulates aggregation territory size by decreasing cyclic AMP pulse size during Dictyostelium discoideum development. Eukaryot Cell 7: 1758–1770. 18676953 10.1128/EC.00210-08 PMC2568059

[33] Graf R (2001) Isolation of centrosomes from Dictyostelium. Methods Cell Biol 67: 337–357. 11550479 10.1016/s0091-679x(01)67023-7

[34] Graf R, Daunderer C, Schliwa M (2000) Dictyostelium DdCP224 is a microtubule-associated protein and a permanent centrosomal resident involved in centrosome duplication. J Cell Sci 113 (Pt 10): 1747–1758. 10769206 10.1242/jcs.113.10.1747

[35] Korenbaum E, Rivero F (2002) Calponin homology domains at a glance. J Cell Sci 115: 3543–3545. 12186940 10.1242/jcs.00003

[36] Goldberg JM, Manning G, Liu A, Fey P, Pilcher KE, et al (2006) The dictyostelium kinome—analysis of the protein kinases from a simple model organism. PLoS Genet 2: e38. 16596165 10.1371/journal.pgen.0020038 PMC1420674

[37] Jorgensen P, Tyers M (2004) How cells coordinate growth and division. Curr Biol 14: R1014–1027. 15589139 10.1016/j.cub.2004.11.027

[38] Kumar A, Molli PR, Pakala SB, Bui Nguyen TM, Rayala SK, et al (2009) PAK thread from amoeba to mammals. J Cell Biochem 107: 579–585. 19350548 10.1002/jcb.22159 PMC2718766

[39] Si K, Lindquist S, Kandel ER (2003) A neuronal isoform of the aplysia CPEB has prion-like properties. Cell 115: 879–891. 14697205 10.1016/s0092-8674(03)01020-1

[40] Majumdar A, Cesario WC, White-Grindley E, Jiang H, Ren F, et al (2012) Critical role of amyloid-like oligomers of Drosophila Orb2 in the persistence of memory. Cell 148: 515–529. 22284910 10.1016/j.cell.2012.01.004

[41] Molli PR, Li DQ, Murray BW, Rayala SK, Kumar R (2009) PAK signaling in oncogenesis. Oncogene 28: 2545–2555. 19465939 10.1038/onc.2009.119 PMC2731678

[42] Rivero F, Dislich H, Glockner G, Noegel AA (2001) The Dictyostelium discoideum family of Rho-related proteins. Nucleic Acids Res 29: 1068–1079. 11222756 10.1093/nar/29.5.1068 PMC29714

[43] Neufeld G, Kessler O (2008) The semaphorins: versatile regulators of tumour progression and tumour angiogenesis. Nat Rev Cancer 8: 632–645. 18580951 10.1038/nrc2404

[44] Vianello F, Olszak IT, Poznansky MC (2005) Fugetaxis: active movement of leukocytes away from a chemokinetic agent. J Mol Med (Berl) 83: 752–763. 16142473 10.1007/s00109-005-0675-z

[45] Bagci T, Wu JK, Pfannl R, Ilag LL, Jay DG (2009) Autocrine semaphorin 3A signaling promotes glioblastoma dispersal. Oncogene 28: 3537–3550. 19684614 10.1038/onc.2009.204

